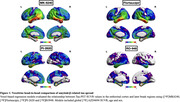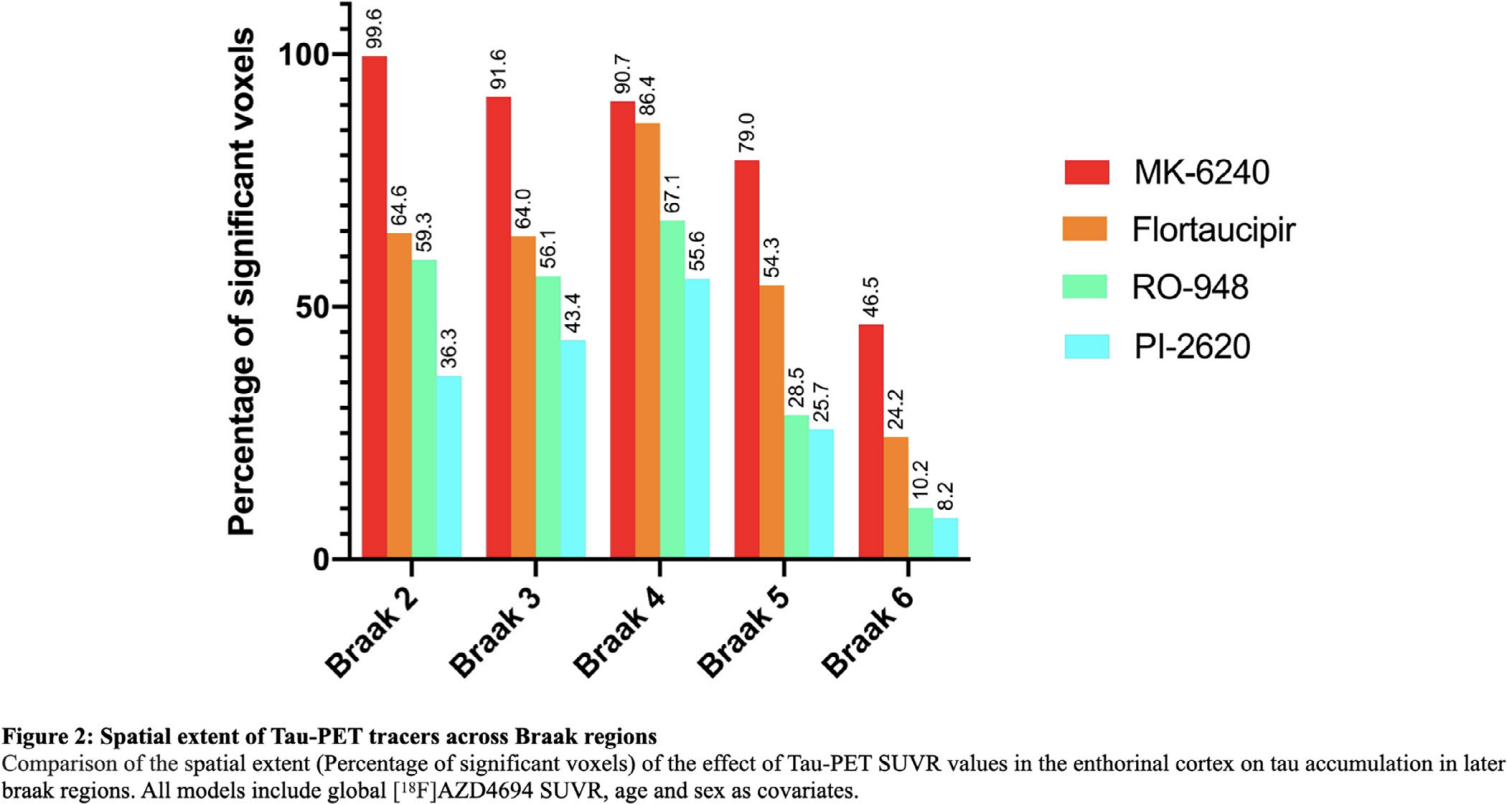# Association between medial temporal and neocortical Tau SUVR across tau imaging agents: HEAD Study

**DOI:** 10.1002/alz.089939

**Published:** 2025-01-09

**Authors:** Nesrine Rahmouni, Cécile Tissot, Seyyed Ali Hosseini, Arthur C. Macedo, Stijn Servaes, Joseph Therriault, Firoza Z Lussier, Jaime Fernandez Arias, Yi‐Ting Wang, Jenna Stevenson, Lydia Trudel, Serge Gauthier, Suzanne L. Baker, Tharick Ali Pascoal, Pedro Rosa‐Neto

**Affiliations:** ^1^ McGill University, Montreal, QC Canada; ^2^ Translational Neuroimaging Laboratory, The McGill University Research Centre for Studies in Aging, Montréal, QC Canada; ^3^ Lawrence Berkeley National Laboratory, Berkeley, CA USA; ^4^ University of Pittsburgh, Pittsburgh, PA USA

## Abstract

**Background:**

The association between medial temporal and neocortical SUVR depends on availability of cortical tau. However, tracer differences in affinity and off‐target binding might interfere in these associations. Here, we examined the association between medial temporal and neocortical SUVR using voxel‐based approach.

**Method:**

We included 92 individuals (46 Cognitively Unimpaired and 46 Cognitively Impaired) from the HEAD Study recruited at McGill University. All individuals were assessed for amyloid‐β (Aβ) deposition using [^18^F]AZD4694‐PET and at least two tau‐PET ligands ([^18^F]MK6240 and [^18^F]Flortaucipir). A subset of 25 individuals had 2 additional tau‐PETs scans: [^18^F]RO948 and [^18^F]PI‐2620. Voxel‐based regression models evaluated the relationship between PET tracers’ uptake in the entorhinal cortex (EC) and its association with the tracer’s uptake in later Braak regions. All models include Aβ‐PET, age, and sex as covariates and RFT was used to account for multiple comparisons. Additionally, we extracted the number of significant voxels in the associations within each Braak regions.

**Result:**

Positive associations were observed between tau‐PET SUVR in the EC (Figure 1). [^18^F]MK6240 EC was correlated with tracer uptake in the whole cortex. [^18^F] Flortaucipir was associated with tracer uptake in similar regions, albeit showing a lower t‐value. [^18^F]PI2620 EC uptake correlated with binding in regions Braak II‐V. Finally, [^18^F]RO948 was associated with uptake in regions Braak II‐IV. We further validated our results by extracting the number of significant voxels in each Braak regions. [^18^F]MK6240 showed the highest percentage of spatial extent of tau spreading in all Braak regions, followed by [^18^F]Flortaucipir and [^18^F]RO948. [^18^F]PI2620 demonstrated the lowest spatial extent.

**Conclusion:**

[^18^F]MK6240 SUVR better predicted the spatial extent of tau spreading in the whole cortex. Results were concordant at the voxel and region‐on‐interested levels. [^18^F]Flortaucipir showed similar results, in lower magnitude. Finally, [^18^F]PI2620 and [^18^F]RO948 were further lower. Nevertheless, the later analyses were conducted in a smaller sample. Our results support the concept that MK6240 affinity possibly explain these results.